# Nutritional strategies against skeletal muscle wasting in cancer-associated cachexia: the role of β-hydroxybutyrate and polyunsaturated fatty acids

**DOI:** 10.1016/j.tranon.2025.102596

**Published:** 2025-11-10

**Authors:** Benjamin Hay, Aurélien Brun, Anne Fougerat, Vera Mazurak, Olivier Le Bacquer, Jérémie Talvas, Frédéric Capel

**Affiliations:** a UMR1019 Unité de Nutrition Humaine (UNH), Université Clermont Auvergne, INRAE, CRNH Auvergne, F-63000 Clermont-Ferrand, France; b Toxalim (Research Centre in Toxicology), INRAE, ENVT, INP-Purpan, UPS, Université de Toulouse, Toulouse, France; c Department of Agricultural, Food, and Nutritional Science, Faculty of Agricultural, Life, and Environmental Science, Division of Human Nutrition, 4-002 Li Ka Shing Centre for Research Innovation, University of Alberta, Edmonton, AB T6G 2R3, Canada

**Keywords:** Protein homeostasis, Myogenesis, Inflammation, Cellular stresses, Ketones, Ketogenic diet, EPA, DHA, Nutrition

## Abstract

Cachexia is a multifactorial syndrome that occurs in many cancers, particularly in their advanced stages, decreasing the quality of life and lifespan of patients. One of the hallmarks of cancer-associated cachexia is skeletal muscle wasting. Multiple causes include inflammation, metabolic deregulation, energy utilization, endoplasmic reticulum and oxidative stress. Loss of skeletal muscle is characterised by an imbalance in protein homeostasis, with decreased anabolism (regulated by the Akt/GSK3/eIF2α and Akt/mTORC1 pathways) and increased catabolism (regulated by autophagy and the ubiquitin-proteasome system), as well as an impairment in myogenesis. Accumulating evidence suggests that dietary intervention of β-hydroxybutyrate, the major ketone body produced by ketogenesis, and n-3 polyunsaturated fatty acids may mitigate skeletal muscle wasting. Polyunsaturated fatty acids and β-hydroxybutyrate are able to favourably modulate inflammation, insulin resistance, unfolded protein response and stresses (such as metabolic stress and oxidative stress). A well-adapted nutritional strategy may include a “classic” diet supplemented with β-hydroxybutyrate and polyunsaturated fatty acids to maintain skeletal muscle integrity and reduce wasting.

## Highlights

• Skeletal muscle homeostasis is closely related to the control of protein anabolism and catabolism.
• Skeletal muscle wasting is highly prevalent in cancer-associated cachexia.
• A combination of ketone bodies and polyunsaturated fatty acids intake may help to preserve skeletal muscle integrity in cancer-associated cachexia.

## Graphical abstract

**Figure:**
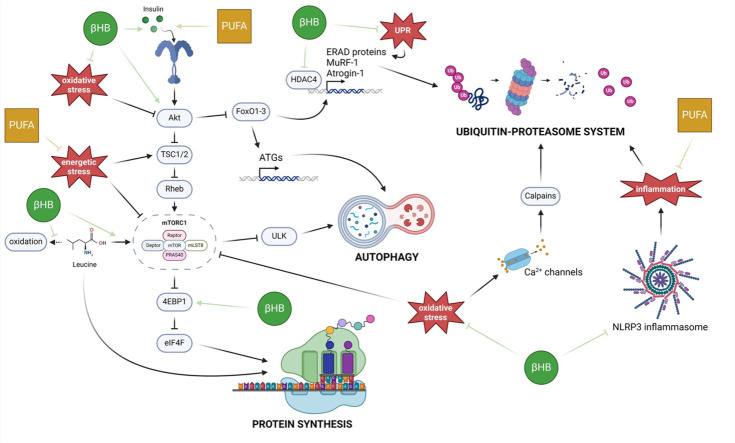

## Introduction

Cancer-associated cachexia (CAC) [1] is a multifactorial syndrome, first described in 1858 [2], induced by numerous mechanisms associated with cancers, notably in their advanced stages, and leading to poor outcomes for patients. A major hallmark of cachexia is weight loss characterized by skeletal muscle (SKM) loss [3] which is closely related to inflammation and altered metabolic processes and having a major impact on survival. The prevalence of the decrease in SKM mass is particularly high in cancers of pancreas, stomach, oesophagus, lung and liver [4]. CAC involves systemic and organ-specific alterations [5] and severe systemic inflammation and insulin resistance were proposed to have a role in the prognosis [6]. Collectively, these symptoms have been shown to decrease both quality of life [7] and lifespan [8].

SKM wasting is characterized by the dysregulation of protein homeostasis and energy metabolism, involving numerous molecular mechanisms, factors and stressors [9]. The phenomenon is related to metabolic dysregulation and systemic inflammation, which worsen the patient’s nutritional state [10]. Systemic inflammation and the decline in SKM quality contribute to frailty, resulting in fatigue, weakness and pain [11]. This leads to significant health complications, decreased recovery from physical inactivity [12], respiratory and cardiac issues [13], and reduced therapeutic efficiency of treatments, including chemotherapy, radiotherapy and immunotherapy (IT) [[14], [15], [16]]. CAC is also a major lethal syndrome for cancer patients, responsible for around 30 % of cancer deaths [17]. Therefore, understanding the metabolic alterations leading to SKM wasting is crucial for developing potential multimodal therapeutic supports for patients.

This review focusses on the control of SKM homeostasis, its dysregulations in the context of CAC and the potential role of β-hydroxybutyrate (βHB) and polyunsaturated fatty acids (PUFAs) in preserving SKM integrity.

## Skeletal muscle homeostasis

### Protein metabolism regulation and myogenesis

**Fig. 1:**
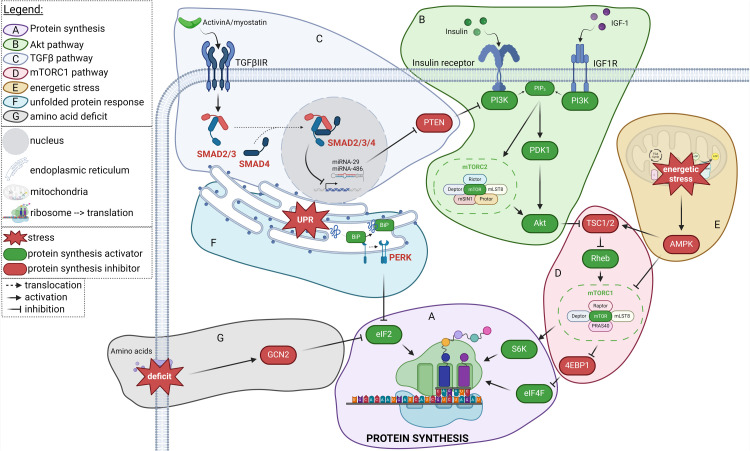
**Protein synthesis pathways in skeletal muscle.** Factors in green are activators of synthesis and factors in red are inhibitors of synthesis. **(A)** The protein synthesis in SKM is mainly driven by S6 kinase and the elongation factors eIF2 and eIF4F. The main activating pathway of protein synthesis is regulated by Akt **(B),** activated by insulin and IGF-1, through PDK1 and mTORC2 and **(C)** inhibited by TGF-β superfamily members (*e.g.*, activin A and myostatin), *via* PTEN. Once activated, Akt inhibits the TSC1/2 complex, allowing mTORC1 activation **(D)**, which leads to the phosphorylation of downstream targets and the initiation of translation through the formation of the eIF4F complex. mTORC1 is also negatively regulated under **(E)** energetic stress, which activated AMPK. The eIF2 complex is inhibited by two other stresses: **(F)** the UPR stress, through activation of PERK, and **(G)** amino acid deficiency activating GCN2.

**Fig. 2:**
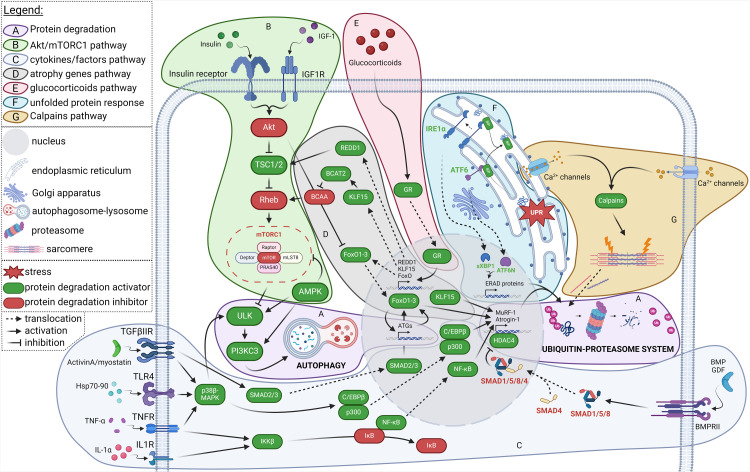
**Protein degradation pathways in skeletal muscle.** Factors in green are activators of degradation and factors in red are inhibitors of degradation. **(A)** Protein degradation in SKM is mainly mediated by autophagy (activated by ULK and PI3KC3 complexes) and the UPS, including the E3 ubiquitin ligases MuRF-1 and atrogin-1. **(B)** Activation of the Akt/mTORC1 pathway by insulin and IGF-1 (see in Fig. 1 for details) decreases the activity of the ULK complex, thereby limiting autophagy. Conversely, autophagy is initiated by **(C)** p38β-MAPK, which is activated by cytokines or factors such as TGF-β superfamily members, Hsp70–90 and TNF- α. These factors, as well as IL-1α, are activators of E3 genes. The expression of these genes, enhanced by HDAC4 activity, is decreased by the SMAD1/5/8/4 complex which is formed and translocated into nucleus after activation by BMP and GDF stimulation. The SMAD2/3 complex, initiated by members of the TGF-β superfamily, activates FoxO1–3, a regulator of autophagy and E3 genes. **(D)** FoxO1–3 is expressed by **(E)** the glucocorticoids pathway, as the Akt/mTORC1 pathway inhibitors, REDD1 and KLF15. **(F)** The UPR activates the expression of ERAD genes via IRE1α and ATF6. ER stress can also **(G)** release Ca^2+^ into the cytoplasm. The increase in intracellular Ca^2+^, supported by voltage-gated Ca^2+^ channels in the SKM membrane, activates calcium-dependent calpains, proteases involved in myofibrillar disruption, which are degraded by the UPS.

After water, proteins are the main component of SKM. Consequently, the maintenance of SKM homeostasis, mass and function is highly related to a tight control of protein metabolism (*i.e.,* anabolic and catabolic processes). A wealth of animal studies provides information on the regulation of SKM anabolism and catabolism. Anabolic signals induce net protein gain and SKM growth. In contrast, catabolic pathways lead to the loss of proteins and organelles. Anabolic and catabolic states are essential during the different phases of development and allows for the renewal of abnormal and damaged proteins. These states are tightly controlled by several interconnected biological processes and enzymatic reactions, detailed below (Fig. 1, Fig. 2).

#### Protein synthesis

As depicted in Fig. 1, SKM’s protein synthesis is mainly controlled by the Akt/mTOR pathway [18] in response to insulin- or IGF-1-related activation of the PI3K protein [19]. PI3K phosphorylates membrane phosphatidylinositol PIP_2_ which activates PDK1 [20] and mTORC2 [21], two activators of Akt.

Akt activates eIF2 and eIF4F complexes to induce mRNA translation into proteins [22]. The eIF2 initiation factor is GTP-dependent and requires the action of eIF2B a nucleotide exchange factor for a full activation [23]. eIF2B is indirectly activated by Akt which inhibits the GSK3 [24,25]. Akt also induces the phosphorylation and dissociation of the TSC1/2 complex, which disables the GTP-binding protein Rheb, allowing mTORC1 activation [26]. In the mTORC1 complex the mTOR kinase is associated to Raptor, differentiating it from mTORC2 in which mTOR is associated to Rictor [27]. mTORC1 can activate S6 kinase, which in turn activates the ribosomal protein S6 of the 40S ribosomal subunit [28]. In parallel, it phosphorylates 4E-BP1 leading to its dissociation from eIF4E, which can then take part in the formation and activation of the eIF4F complex to initiate mRNA translation by recruiting the 40S ribosomal subunit [29].

#### Protein degradation

The degradation of proteins in SKM involves two main mechanisms: autophagy and the ubiquitin-proteasome system (UPS) (Fig. 2).

##### Autophagy

Autophagy plays a crucial role in maintaining SKM homeostasis, by degrading non-essential and recycling damaged proteins and amino acids, thereby limiting SKM stress and acute damage [30,31].

Autophagy can be induced by intracellular and extracellular factors, such as amino acid deficiency, inflammation, oxidative stress, and mTOR inhibitors [32,33]. The process is characterized by the formation of autophagosomes, which then fuse with lysosomes to form autophagolysosomes able to degrade proteins by lysosomal proteolytic enzymes [34]. When recycled amino acids reach a sufficient level, they can activate mTORC1 to stimulate cell growth and proliferation [35]. Detailed reviews focused on the autophagy process are already available [31,36]. Briefly, initiation of autophagy is driven by the ULK1 complex. The autophagosome is then formed through the maturation of the phagophore, which is facilitated by the PI3KC3 complex and autophagy genes such as ATGs. In SKM, these complexes and genes are mainly regulated by AMP kinase (AMPK) [37], mTORC1 [38] and FoxO [39]. ULK1 activating phosphorylation and autophagy-related genes expression are also induced by C/EBPβ/p38β-MAPK pathway [40].

##### Ubiquitin-proteasome system

The UPS operates through an enzymatic cascade that results in the ubiquitination of proteins, followed by their degradation by the 26S proteasome [41]. This reaction involves three types of enzymes. Ubiquitin-activating enzymes (or E1 enzymes) activate ubiquitin, which can then be transferred to the ubiquitin-conjugating enzyme (or E2 enzymes). Finally, ubiquitin-ligase enzymes, also calledE3 enzymes enable serial ubiquitination reactions, resulting in the attachment of an ubiquitin chain to target proteins [42]. Among these enzymes, two are primarily involved in the degradation of SKM proteins: MuRF-1 (*Trim63*) and MAFbx/Atrogin-1 (*Fbxo32*) [43]. MuRF-1 specifically targets sarcomere proteins [44], which are essential for the structure and contractility of SKM. Atrogin-1 targets MyoD, a regulator of myogenesis, and eIF3F, a key player in protein synthesis [45]. E3 genes are regulated by many factors, such as, the transcription factors FoxO and KLF15 which can induce the transcription of MuRF-1 and Atrogin-1 [46,47]. FoxO proteins are then a crucial regulators of protein degradation. Their expression is increased by activation of the SMAD2/3 pathway [48].

Other SMADs, SMAD1/5/8/4, are negative regulators of the UPS [49]. When activated, the SMAD1/5/8 complex recruits SMAD4. The SMAD1/5/8/4 complex translocates into nucleus and inhibits HDAC4, an epigenetic enhancer of E3 enzyme genes, resulting in the inhibition of E3 gene expression. Inflammation could also increase protein degradation through the activation of NF-κB, which notably increases the expression of MuRF-1 [50].

#### Myogenesis

A negative protein balance induces atrophy within a dynamic and complex system, influenced not only by protein homeostasis, but also by various microenvironmental factors that collectively modulate its mass. The interplay between extracellular signals, including growth factors and cytokines, and neuro-mechanical stimuli, regulates the activation and differentiation of SKM stem cells, also called satellite cells [51]. These adult quiescent cells, expressing the transcription factor Pax7, are located closely to SKM fibres, specifically between the basal lamina and the sarcolemma (cell membrane) of the fibres [52]. Upon acute injury, satellite cells are rapidly activated in response to inflammatory signals, including IL-6, TNF-α, IL-1, IL-4 and IL-10 [53]. These cells then undergo proliferation, differentiation and fusion, which are crucial for replacing damaged SKM fibres and restoring SKM structure and function. However, chronic injury or pathological conditions disrupt the balance between SKM regeneration and degeneration leading to impaired myogenesis and SKM wasting [54]. This impairment triggers the recruitment of fibroblasts, leading to fibrosis [55].

## Skeletal muscle homeostasis dysregulations in cancer-associated cachexia

SKM wasting is a major hallmark of CAC. The loss of SKM mass is strongly related to the imbalance between protein synthesis and degradation. Several factors or biological processes have been identified as key contributor to this imbalance and will be discussed here.

### Molecular and energetic factors

Most of the factors affecting SKM homeostasis in the context of CAC are endogenous factors that are directly secreted by the tumor. Indeed, cancers are characterized by the deregulation of various metabolic, inflammatory, hormonal, and growth factors along with the secretion of tumor-derived factors. Collectively, these factors negatively impact the pathways responsible for maintaining SKM homeostasis by acting as upstream regulators at the membrane, thereby initiating a cascade of molecular events (Fig. 1, Fig. 2).

#### Inflammation

Cancer cells exhibit abnormal growth and secretions that trigger immune system activation and the release of pro-inflammatory cytokines, leading to a systemic inflammatory state [56]. Although, tumors adapt to their microenvironment to escape immune detection [57], cytokines and signalling molecules are still released by the host immune system, further intensifying acute inflammation which in turn disrupts the metabolism and homeostasis of distal tissues, including SKM [58]. Among the factors released by tumor cells, the most significant for SKM protein homeostasis include members of the TGF-β superfamily (such as activin A and myostatin), Hsp70 and Hsp90, TNF-α and interleukins (*e.g.,* IL-1α and IL-6) [59]. These secreted factors promote protein degradation by inducing autophagy or upregulating E3 genes expression (Fig. 2). By contrast, bone morphogenic protein (BMP) and growth differentiation factor (GDF) can decrease the expression of E3 genes through SMAD1/5/8/4 pathway (Fig. 2). However, IL-6 and activin A activate Noggin, which inhibits BMP [60]. In parallel, members of the TGF-β superfamily can also reduce protein synthesis through the SMAD2/3/4 pathway (Fig. 1) and inhibiting Akt *via* PTEN [[61], [62], [63]]. Moreover, systemic inflammation that develops during CAC further disrupts hormone secretion and induces oxidation stress (described below).

#### Metabolic dysregulation

**Fig. 3:**
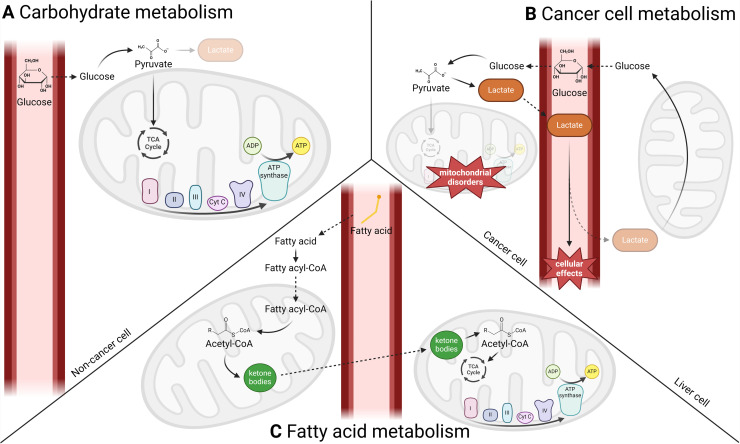
**Carbohydrate, FA and tumor metabolisms. (A)** The most common diet for healthy people consists mainly of carbohydrates, made up glucose. In cells, glucose is metabolized to pyruvate (glycolysis), which is mostly converted to acetyl-CoA in the mitochondria. Acetyl-CoA supplies the TCA cycle, supplying mitochondrial respiratory chain for ATP production. **(B)** In cancer cells, mitochondrial dysfunctions prevent the conversion of pyruvate to acetyl-CoA to promote lactate production (lactic fermentation). Lactate can diffuse into liver cells where it supplies gluconeogenesis to produce glucose. Lactate metabolism, through the Cori cycle, has a lower energy yield (ATP) but can be used for glucose regeneration in the liver (gluconeogenesis) or for important cellular effects leading to tumorigenesis. **(C)** In the liver mitochondria, FAs are metabolized to acetyl-CoA (FA β-oxidation), which is converted to KBs (ketogenesis). Acetoacetate and βHB are distributed in the bloodstream to extrahepatic tissues and converted to acetyl-CoA (ketolysis) which enters the TCA cycle to produce ATP.

Unlike normal cells which typically generate energy through the tricarboxylic acid (TCA) cycle and oxidative phosphorylation (OXPHOS) in the mitochondria, most cancer cells rely on anaerobic glycolysis for energy production, a process known as the Warburg effect [64] (Fig. 3). This metabolic shift results in increased lactate production [64]. While lactate is essential for tumor development [65], it also initiates gluconeogenesis via the Cori cycle [66] (Fig. 3). Through these modulations, cancer leads to metabolic adaptations disrupting insulin signalling and glucose homeostasis [67]. Insulin resistance and compensatory hyperinsulinemia, together with systemic inflammation, are key contributors to CAC and have been associated with poor outcomes [6]. Insulin resistance disrupts protein homeostasis of the muscle by shifting the balance toward degradation, as the rate of protein synthesis can no longer compensate for the increased rate of protein breakdown (Fig. 1, Fig. 2) [67,68]. Inflammation can also decrease the secretion of IGF-1 by the liver [69] and further aggravate the alteration in protein anabolism due to a decreased activation of the Akt/mTOR pathway.

Other hormones, such as glucocorticoids, also play a role in the dysregulation of protein homeostasis in CAC, through the glucocorticoids receptor pathway (Fig. 2) [70] or suppression of anti-tumor immunity [71]. The production of glucocorticoids is increased by the activation of the hypothalamic-pituitary-adrenal axis which is related to the systemic inflammation [70]. An imbalance in the secretion of glucocorticoids could also be involved in an impaired metabolic adaptation, that favours SKM loss [72].

The different metabolic alterations affecting the mitochondria (biogenesis, fusion/fission ratio, activity, mitophagy and oxidative stress defence) affect SKM in many ways. These are well described and summarized in Table 1.

**Table 1:** Mitochondrial disruptions and their consequences on skeletal muscle in cancer-associated cachexia context.

Metabolic alterations	Mitochondria defects	Consequences in SKM	References
Biogenesis	PGC-1α, NRF and TFAM downregulation	reduced mitochondria content; reduced ATP production; impaired myogenesis/regeneration	[[73], [74], [75], [76]]
	PGC-1α downregulation	switch from type I to type II muscle fibres	
Fusion	MFN-1, MFN-2 and Opa1 downregulation	reduced mitochondrial DNA; elevated ROS; reduced ATP production	[[73], [74], [75], [76], [77]]
Fission	FIS-1, Dnm1l and Oma1 upregulation	proapoptotic signals; elevated ROS; reduced ATP production; AMPK activation	[[73], [74], [75], [76], [77]]
Activity (OXPHOS)	decreased MRC; proton leak (UCPs); dysregulated TCA cycle	reduced ATP production; impaired myogenesis/regeneration	[[73], [74], [75], [76],^78^,^79^]
Mitophagy	Bnip3 and LC3 pathways	AMPK, FOXO, mTORC1 signalling disruption	[[73], [74], [75], [76],^78^,^80^]
Oxidative stress	ROS defence proteins downregulation	oxidative stress	[73,75,76,80]

### Cellular stresses

CAC is associated with metabolic stress, amino acid and energetic deficiencies [81]. Endoplasmic reticulum (ER) stress, unfolded protein response (UPR) [82] and oxidative stress [83] are also activated. These features further exacerbate the loss of SKM protein and then SKM wasting (Fig. 1, Fig. 2).

#### Amino acid and energetic deficiencies

Amino acid deficiency, in part linked to malnutrition, induces GCN2 kinase activation, leading to a reduction in protein synthesis through the phosphorylation of eIF2α [84] (Fig. 1). Energy stress occurs when metabolic dysregulations impair ATP synthesis. It elevates the AMP/ATP ratio resulting in the activation of AMPK [85]. This kinase phosphorylates the TSC1/2 complex, enhancing its inhibitory activity on Rheb [86], and phosphorylates Raptor protein from the mTORC1 complex [86]. Therefore, AMPK drives the inhibition of mTORC1 and represses protein synthesis (Fig. 1). Mitochondrial abnormalities such as reduced biogenesis and dynamic are consistently observed in cachectic muscle and may contribute to the reduction in ATP production [87]. Activation of AMPK modulates autophagy, redox homeostasis, and cell survival pathways, thereby protecting against oxidative stress and inflammation [88]. However, persistent or chronic activation of AMPK may be detrimental [89].

#### Endoplasmic reticulum stress

In SKM, ER stress can induce two important responses that promote imbalance in protein homeostasis: the UPR and the liberation of Ca^2+^ in cytosol (Fig. 1, Fig. 2).

The UPR drives the activation of PERK, ATF6 and IRE1α pathways by removing the ER chaperone BiP from their receptors located in ER membrane [90]. After release and dimerization, PERK phosphorylates the eIF2α subunit [91], inducing the inhibition of eIF2B activity and thus the protein synthesis (Fig. 1). ATF6 and IRE1α are involved in ER-associated protein degradation (ERAD), initiated by the presence of misfolded proteins in the cell [92]. Then, ER stress causes BiP dissociation from ATF6, which migrates in the Golgi apparatus for cleavage and liberation of its N-terminus part (ATF6N), which then translocates to the nucleus [93]. The UPR also releases IRE1α, allowing its activation through oligomerization and autophosphorylation [94]. Activated IRE1α complex exhibits RNase activity leading to specific splicing of *Xbp1* mRNA and the synthesis of sXBP1 isomer [95]. ATF6N [93] and sXBP1 [96] are transcription factors that induce the expression of ERAD genes and activate the UPS (Fig. 2).

Offer stress-related Ca^2+^ released in cytosol lead to the activation of calpains [97]. These enzymes are calcium-activated proteases and are involved in the disruption of myofibrils by cleaving and depolymerizing myofibrillar components, such as desmin [98] or titin [99]. The sarcomere proteins, released in the form of monomers, can then be taken up by the UPS for degradation (Fig. 2).

#### Oxidative stress

Oxidative stress is defined as an imbalance in the production of free radicals and the ability to detoxify these molecules, in favour of reactive species, particularly reactive oxygen species (ROS) and reactive nitrogen species (RNS) [100]. In SKM, ROS are notably produced in response to the activation of the TNF-α/TNF1R pathway, which initiates a signalling cascade activating phospholipase A2 and NAD(P)H oxidase [101] and could further aggravate mitochondrial functions during CAC and amplify ROS production [83]. The generation of RNS is also initiated by cytokines, such as TNF-α, via iNOS expression [101]. Oxidative stress impairs SKM protein homeostasis, particularly by affecting the Akt/mTORC1 pathway and intracellular Ca^2+^ levels [101].

#### Myogenesis impairment

Multiple lines of evidences indicate that CAC disrupts the myogenic program [54,102]. These defects are primarily related to aberrant overexpression of Pax7 in satellite cells triggered by a sustained pro-inflammatory environment. Prolonged Pax7 expression compromises the commitment of SKM stem cells to differentiation programming, leading to inadequate myofiber regeneration [103]. One significant alteration is the downregulation of IGF-1 signalling [104], which is essential for SKM growth and repair. Additionally the myostatin/activin A/TGFβIIR pathway becomes hyperactivated [105], further inhibiting SKM growth while promoting protein degradation (Fig. 1, Fig. 2). Collectively, these molecular disruptions result in a marked loss of SKM mass and function, which not only diminished patients’ quality of life but also impairs their ability to tolerate and respond effectively to anti-cancer therapies.

## Skeletal muscle homeostasis preservation

As CAC is a multifactorial disorder [106], a preventive or therapeutic approach must consider multimodal interventions [[107], [108], [109]], including pharmacological, nutritional, and other lifestyle (*e.g.* exercise, social connections or risks factors such as smoking or alcohol consumption) factors. Energy and protein are required to sustain anabolism and limit catabolism. Nutrients can also serve as signals and substrates for SKM. Some amino acids, PUFAs and vitamins, have been identified as stimulators of protein synthesis in SKM [[110], [111], [112]]. Specific dietary approaches using these compounds could be proposed to tackle the molecular and biological alterations related to CAC. Additionally, recent research has explored the potential of the ketogenic diet (KD) and βHB, the primary ketone body (KB) produced during this diet, against cancer, but the effect on CAC and SKM homeostasis remains poorly described. Given that a KD consists of increased amounts of dietary fats, it is also interesting to investigate how different types of dietary fatty acids (FAs) interfere with CAC.

### β-hydroxybutyrate

#### Energy source and physiological effects

In healthy individuals consuming a well-balanced diet, carbohydrates and FAs serve as the primary energy sources for metabolism during the fed and fasting states, respectively (Fig. 3). During prolonged fasting, the use of fat for energy becomes predominant (Fig. 3) as glycogen stores are reduced and gluconeogenesis decreased to prevent muscle wasting [113]. During fasting, FAs, derived from adipose tissue lipolysis, are taken up by the liver and converted into fatty-acetyl-CoA in the hepatocyte mitochondria by FA β-oxidation. When acetyl-CoA levels accumulate in liver mitochondria, they are converted to KBs, including acetoacetate, βHB and acetone. KBs are released by the liver into the circulation and transported to other tissues where they undergo ketolysis to produce acetyl-CoA, fuelling the TCA cycle to produce ATP. The KD biochemically resembles fasting, promoting fat breakdown, production of KBs, lowering blood glucose and insulin levels [114].

In addition to being an energy source, βHB might also be involved in cellular signalling, post-transcriptional modifications, inflammation, oxidative stress and lipid synthesis [[115], [116], [117], [118]]. In a recent study, Zhou et *al.* [119] have shown, in a mouse model of colon CAC, that a higher serum level of βHB (induced by subcutaneous injection of ethyl 3-hydroxybutyrate), prevents loss of body weight and fat, decreases SKM wasting, reduces tumor weight, and increases survival rates. They also report a reduction in circulating inflammatory factors and improved antioxidant capacity in cachectic mice receiving ethyl 3-hydroxybutyrate, suggesting that βHB may have both anti-cancer and anti-CAC effects.

#### Molecular effects of βHB on protein metabolism

**Fig. 4:**
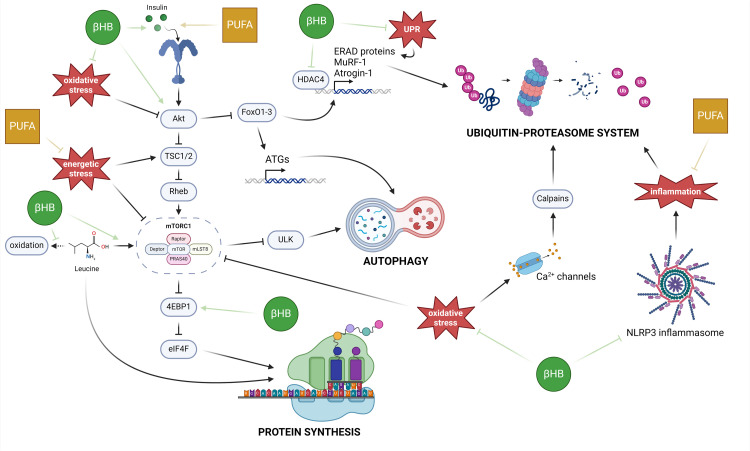
**Regulations of protein homeostasis pathways by β-hydroxybutyrate and polyunsaturated fatty acids.** βHB and PUFAs can restore SKM protein homeostasis by regulating anabolic and catabolic systems at different levels. The KB modulate some of the most important stresses involved in SKM wasting (inflammation, UPR and oxidative stress), whereas PUFAs regulate insulin sensitivity, metabolic efficiency and inflammation. Inflammation is reduced by inhibiting the NLRP3 inflammasome and limiting IL-1β secretion. To regulate the UPR and oxidative stress, βHB acts as a regulator of gene expression, by decreasing the expression of UPR genes and increasing the expression of antioxidant genes. In addition, the E3 gene expression can be reduced inhibiting HDAC4 activity. The anti-catabolic effect of βHB, via the decrease in E3 and ATGs gene expression, can also be explained by the activation of the Akt/FoxO3a pathway through the activating phosphorylation of Akt and the increase in insulin sensitivity, supported by PUFAs. This increase in protein anabolism is supported by the inhibition of 4E-BP1 and the activation of leucine-mediated protein synthesis. βHB increases the activation of mTORC1 by leucine while limiting the oxidation of this essential amino acid and increasing its incorporation into SKM proteins. In addition, the increase in metabolic efficiency provided by PUFAs restores the AMP/ATP ratio, limiting the inactivation of mTORC1 by AMPK, and therefore increasing protein synthesis.

At the molecular level, βHB has beneficial effects against the deregulation of protein homeostasis [120]. These effects include direct modulation of gene expression [121], regulation of Akt/FoxO3a pathway [120], enhancement of leucine/mTORC1-mediated protein synthesis [122] and modulation of inflammation [123,124]. βHB can thus preserve SKM by modulating both protein synthesis and degradation (Fig. 4). The regulation of gene expression by βHB facilitates the downregulation of the UPS (Fig. 4) through the inhibition of enhancers of E3 gene expression [121], such as HDACs, the reduction of UPR- and oxidative stress-related gene expression [120], decreased activation of calpains, and restoration of the Akt/mTORC1 protein synthesis pathway [101] (Fig. 4). Additionally, βHB enhances insulin sensitivity [123] and promotes Akt phosphorylation, leading to the downregulation of protein breakdown mediated by FoxO3a and mTORC1 [120] (Fig. 2). βHB also activates protein synthesis by increasing 4E-BP1 phosphorylation [120] and reducing leucine oxidation, promoting leucine-dependant mTORC1 [122,125] (Fig. 4).

Regarding its effects on the myogenic program, a study on equine satellite cells found that β‑hydroxy-β-methylbutyrate enhances cell survival and protects against oxidative stress induced by hydrogen peroxide [126].

In addition, it has been demonstrated that tumor-derived IL-6 suppresses PPARα, one of the major regulators of hepatic ketogenesis [71]. Under caloric deficiency, this results in an increase of glucocorticoid secretion and a decrease of anti-tumor immunity [71]. Therefore, higher βHB intake may limit glucocorticoid accumulation and restore anti-tumor activity.

### Polyunsaturated fatty acids

The quality of dietary FAs has a major influence on human health and health effects of PUFAs are now well recognized [127]. There are two classes of PUFAs, n-3 (α-linolenic acid (ALA), eicosapentaenoic acid (EPA) and docosahexaenoic acid (DHA)) and n-6 (linoleic acid (LA) and arachidonic acid (ARA)) PUFAs. n-3 PUFAs are anti-inflammatory and have been shown to reduce tumor growth, as well as enhance tumor response to some therapies *in vitro* and clinical studies [128]. Provision of n-3 PUFA through diet therefore impacts both muscle and tumor to benefit the host in CAC. n-6 PUFAs are precursors of pro-inflammatory mediators. A high n-6 to n-3 ratio (often 15:1 to 20:1), as typically observed in Western diets, is linked to increased risk of cardiovascular disease, obesity, cancer, inflammatory, autoimmune, and mood disorders. A single intervention with healthy PUFAs can have multiple effects on both tumors and “normal” tissues simultaneous leading to an improvement of CAC patient’s health [129]. Despite being studied for over 25 years, no formal recommendations for n-3 PUFA exist and more clinical intervention are required to define the best form of intake, doses and duration of supplementation.

#### Modulation of immunotherapy by polyunsaturated fatty acids

IT, like other treatments, is less effective in individuals with CAC [16,130,131]. IT applies blocking antibodies, such as anti-PD1 or anti-PDL1, prevents the tumor from inhibiting the activation of CD8+ cytotoxic T lymphocytes [57]. Activation of CD8+ cytotoxic T lymphocytes allows the secretion of IFN-γ, involved in suppression of tumor growth, notably by triggering ferroptosis [132], a non-apoptotic cell death mechanism [133]. Ferroptosis is induced by the peroxidation of lipids, notably PUFA-containing phospholipids [128,134]. This mechanism is increased by formation of PUFA-accumulation lipid droplets [128] in the micro-environment of cancer cells, increasing the availability of PUFAs and the activation of ferroptosis regulators such as GPX4, SLC7A11 and ASCL4 [134]. Through this specific accumulation, it has been speculated that PUFAs improve IT by increasing ferroptosis specifically in cancer cells, suggesting that a targeted nutritional approach with increased PUFAs intake could be promising for CAC patients to enhance response to IT.

#### Molecular effects of PUFAs on protein metabolism

FAs are important molecules for many biological processes such as metabolism, gene expression, response to hormones or cell structures through membranes composition [127]. The protection of SKM loss by n-3 PUFAs [135,136] could be related to several factors, such as an improved insulin sensitivity [137], attenuation of inflammation [138] and an improvement of nutritional status [139]. It results in an optimized protein homeostasis (Fig. 4) as insulin sensitivity and inflammation are master regulators of protein anabolism and catabolism, respectively (Fig. 1, Fig. 2). Additionally, DHA and EPA increase GLUT-4 expression, the main glucose transporter in SKM, and the number of mitochondria in SKM cells [140], both facilitating a more efficient utilisation of glucose as an energy substrate, which result in a decreased AMP/ATP ratio and a better metabolic efficiency. This, in turn, restores mTORC1 activity and protein synthesis limiting AMPK activation (Fig. 1, Fig. 4).

PUFAs have also been implicated in the myogenic program of satellite cells [141]. Studies on the effects of PUFA supplementation, particularly EPA and DHA, have shown conflicting results. In one study, supplementation with EPA and DHA induced trans-differentiation from myoblasts to an adipogenic phenotype [142], indicating a potential diversion of satellite cells from their normal myogenic lineage towards fat cell formation. Conversely, other studies have demonstrated that EPA and DHA can upregulate master SKM differentiation genes, such as MyoD, promoting the differentiation of satellite cells into myotubes [143,144]. This upregulation suggests that, under certain conditions, PUFAs may enhance SKM regeneration and support the myogenic program of satellite cells. These seemingly contradictory findings highlight the complexity of PUFA effects on SKM biology and timing of exposure during the differentiation process, doses applied, length of time *etc*.

### Nutritional intervention targeting β-hydroxybutyrate and polyunsaturated fatty acids

Accumulating evidence has shown that βHB and PUFAs could be promising molecules for the maintenance of SKM homeostasis during CAC. They may exert their effects through direct effects on SKM or by limiting tumor progression or activity. Moreover, supplementation of βHB and PUFAs both showed a potentiating effect on IT efficacy. βHB-mediated antineoplastic effect that relies on T cell-mediated cancer immunosurveillance [130] and PUFAs enhance ferroptosis of tumor cells induced by immune cells reactivated by IT [128,134].

#### Ketogenic diet

Endogenous biosynthesis of βHB can be achieved through a KD. However, in the context of CAC the potential benefits of the KD are controversial [145]. KD was first described in 1925 by Peterman et *al.* [146]. It is defined by a very high fat, very low carbohydrate, and moderate protein intake. Typically, KD provides approximately 70–90 % of total calories from fat, 6–20 % from protein, and 4–10 % from carbohydrates. This composition shifts the body’s metabolism from glucose to fat-derived KB as the primary energy source [114,123,130], while providing higher levels of PUFAs [114]. In mice, many beneficial effects have been associated to KD feeding. For example, KD has been shown to extend longevity and health span in mice [147,148]. Furthermore, evidence from rodent models and human studies indicates that KD may also improve anti-tumor responses to cancer therapy [149,150].

Few clinical studies have evaluated the potential benefits of KD on CAC and consistently observed no serious adverse events or toxicity [149]. Beneficial effects, included improvements in quality of life, body composition, and metabolic parameters, were reported in different cancer studies [[151], [152], [153], [154]]. Meta-analysis showed that quality of life and survival, but not body weight, could be improved by n-3 PUFAs [155] and that these FAs may be protective against weight loss in patients with non-small cell lung cancer [156]. However, the clinical trials supporting the beneficial effects of KD or n-3 PUFAs were conducted with small sample size and not always randomized. Furthermore, there was substantial heterogeneity in the criteria used to define CAC, chemotherapy and supply of n-3 PUFAs. More clinical evidence is then necessary to address if the improvement in the lipid profile of KD could help patient management and tolerance to treatments.

Weight loss that occurs on a KD has recently been attributed in part to increased circulating GDF15 [157]. Yet, plasma levels of GDF15 in cancer patients correlate with CAC and reduced survival [158,159]. Although some studies have reported beneficial effects of KD on CAC [160], others have led to conflicting results, showing a negative effect on CAC [72,161] and SKM homeostasis [162]. In CAC models, a KD accelerated SKM wasting through a reduced corticosterone level leading to impaired adaptation to stress [72] or through possible alterations in protein homeostasis [161]. Differences between study outcomes are observed in the literature (and reviewed by Yakupova et *al.* [162] in 2022), in mouse models, including higher insulin-resistance, increased corticoids levels, upregulation of UPS system genes, SKM remodelling, increased mitochondrial ROS production, accumulation of intramuscular triglycerides and enhanced lipid oxidation [162]. These discrepancies likely reflect differences in experimental conditions (*e.g.*, the models, the age, the health conditions and exposure or pathology), as well as variation in KD composition (notably the degree of FA saturation) and duration [162]. In addition, KD has been shown to induce cellular senescence in multiple organs, including the heart and kidney [163], and long term KD feeding could impair insulin secretion [164] and promote hepatic steatosis and inflammation in mice [165]. This nutritional approach is not well optimized in clinical practice and presents obvious risks and difficulties for patients. Indeed, adherence to the KD was generally low and patients with CAC, often experience several nutritional symptoms that reduce food intake, which could be further exacerbated by the initiation of a KD [166]. Moreover, sex-specific responses to KD have been reported [167] and may contribute to the heterogeneity of outcomes observed across studies.

#### βHB supply

For translation to humans, following a KD during the trajectory of a cancer diagnosis and cachexia poses challenges, therefore mimetics of the KD, such as βHB provide an alternative approach. Considering the controversies, risks and the low long-term compliance associated with KD, an optimized supply of βHB, the major KB produced during a KD, within a “conventional” diet, enriched in n-3 PUFAs, could be sufficient to support CAC therapies and management in clinical practice. It has been suggested that n-3 PUFAs may promote ketogenesis and metabolic flexibility during caloric restriction in obese women [168], but to the best of our knowledge, no study has specifically investigated the impact of the combination of βHB and PUFAs on SKM. βHB was tested in a multifactorial mouse model of CAC syndrome to study the role of KBs in attenuating SKM atrophy [169]. Injection of a ketone diester, increased circulating βHB levels, and attenuated CAC (at the level of tumor burden, SKM atrophy and comorbidities) through the restoration of appetite and the decrease of inflammatory syndrome [169].

The most significant clinical limitation of βHB supplementation is the risk of gastrointestinal distress and ketoacidosis, particularly in patients with diabetes or at risk of chronic kidney disease [[170], [171], [172]]. The use of n-3 PUFAs does not led to major side effects at doses below 2 g/day. These include low risk of nausea, unpleasant taste, gastrointestinal discomfort.

#### Research gaps and priorities

Most clinical evidence on the effects of increasing circulating KBs against CAC comes from small, non-randomized, or single-arm studies. The outcomes appear to vary depending on cancer type, sex, and diet composition. More research, especially in humans, is needed to clarify their therapeutic potential.

## Conclusion

SKM is one of the most affected organs in many cancers, with a significant loss of mass and function, associated with CAC syndrome. This SKM wasting is one of the most important cause of mortality and CAC is associated with a reduction in the efficacy of therapies. It is therefore necessary to develop therapeutic strategies in order to limit cancer progression and protect the SKM. However, CAC is a multifactorial syndrome, affecting a broad range of cellular pathways and little is known about the mechanisms in humans.

It would be too simplistic to apply a single therapy against CAC. However, combining strategies could be a promising approach to reduce tumor activity and preserve SKM. For example, enhancing IT effectiveness, such as by inducing ferroptosis through increased intake of PUFAs, could be a treatment option. Furthermore, PUFAs have demonstrated beneficial effects on SKM, notably by reducing inflammation and metabolic stress and these effects could be supported by the βHB. These observations suggest that a combination of IT and an increased intake of PUFAs and βHB could be an attractive strategy for patients suffering from CAC.

There are various methods to increase PUFAs and βHB intake (*e.g.*, dietary supplementation). However, it is crucial to await further results from pre- and clinical research before widely recommending the KD or βHB as a CAC treatment. Interestingly, serum levels of βHB upon KD feeding oscillate in a circadian manner, revealing the complexity and the challenge to better understand the effects of this metabolite [173]. Intermittent KD has recently emerged as a potential alternative which could be more beneficial and easier for patients than a long-term continuous KD [163,174].

In conclusion, current therapies for CAC are under-developed and anti-cancer treatments are not well adapted for patients. However, with current and potential future discoveries, it may be possible to develop more efficient and safer therapeutic monitoring. Regarding the molecular targets of KBs and n-3 PUFAs, a combination of targeted therapy, optimized nutrition (through KD or targeted supplementation) could be promising against CAC. This therapeutical approach could also benefit from incorporating physical activity, which has shown beneficial effects in the protection of SKM and the stimulation of anabolic signals [175,176], as well as in tumoral progression and protection of CAC [177]. Finally, other opportunities exist such as the modulation of microbiome (in particular gut microbiota) [[178], [179], [180]] and should also be explored.

## Funding

This work was supported by France Canada Research Fund (FCRF); a grant given by the “SFNCM” scientific society as part of the 2021 French national young researcher contest to A.B.; Clermont-Auvergne-Metropole for the support of A.B.’s PhD; French Ministry of Higher Education and Research (MESR) for the support of B.H.’s PhD; Mitacs Globalink Research Award (GRA) program 2024; a research grant from Groupe Lipides Nutrition (GLN).

## Declaration of interest statement

All authors declare that they have no actual or potential conflict of interest including any financial, personal or other relationships with other people or organisations within that could inappropriately influence their work.

## Abbreviations

4E-Binding Protein 1, 4E-BP1; Alpha-Linolenic Acid, ALA; AMP-activated protein Kinase, AMPK; Arachidonic Acid, ARA; Achaete-SCute family bHLH transcription factor 4, ASCL4; Activating Transcription Factor 6, ATF6; AuTophagy-Related Gene, ATG; Branched-Chain Amino Acid, BCAA; Branched Chain Amino acid Transaminase 2, BCAT2; Binding immunoglobulin Protein, BiP; Bone Morphogenetic Protein, BMP; BMP Receptor type-II, BMPRII; Bcl-2 interacting protein 3, Bnip3; CCAAT-Enhancer-Binding Proteins, C/EBP; Cancer-Associated Cachexia, CAC; Cluster of Differenciation, CD; DocosaHexaenoic Acid, DHA; Dynamin-1-like, Dnm1l; Ubiquitin-activating, E1; Ubiquitin-conjugating, E2; Ubiquitin-ligase, E3; eukaryotic translation Initiation Factor, eIF; EicosaPentaenoic Acid, EPA; Endoplasmic Reticulum, ER; Endoplasmic-Reticulum-Associated protein Degradation, ERAD; Fatty Acid, FA; F-box only protein 32, Fbxo32; Mitochondrial FISion 1, FIS-1; Forkhead box protein O, FoxO; General Control Nonderepressible 2, GCN2; Growth Differenciation Factor, GDF; GLUcose Transporter type 4, GLUT-4; Glutathione PeroXidase 4, GPX4; Glucocorticoids Receptor, GR; Glycongen-Synthase Kinase 3, GSK3; Histone DeACetylase, HDAC; Heat shock proteins, Hsp; InterFeroN-g, IFN- g; Insulin-like Growth Factor-1, IGF-1; IGF1 Receptor, IGF1R; Inhibitor of nuclear factor Kappa-B Kinase subunit b, IKKb; InterLeukine, IL; IL1 Receptor, IL1R; inductible Nitric Oxide Synthase, iNOS; endoplasmic reticulum to nucleus signalling 1, IRE1 a; ImmunoTherapy, IT; Inhibitor of nuclear factor kB, IkB; Ketone Body, KB; Ketogenic Diet, KD; Krueppel-Like Factor 15, KLF15; Linoleic Acid, LA; microtubule-associated proteins 1A/1B Light Chain 3, LC3; Muscle Atrophy F-box, MAFbx; Mitogen-Activated Protein Kinase, MAPK; MitoFusiN, MFN; Mitochondrial Respiratory Chain, MRC; mammalian Target Of Rapamicin Complex, mTORC; Muscle RING?Finger protein 1, MuRF-1; Myoblast Determination, MyoD; Nuclear Factor K-light-chain-enhancer of activated B cells, NF-kB; NOD-Like Receptor family, Pyrin domain containing 3, NLRP3; Nuclear Respiratory Factor, NRF; Optic atrophy 1, Opa1; OXidative PHOSphorylation, OXPHOS; Paired box 7, Pax7; Programmed cell Death protein 1, PD1; Pyruvate Dehydrogenase Kinase 1, PDK1; Programmed cell Death-Ligand 1, PDL1; Protein kinase R-like Endoplasmic Reticulum Kinase, PERK; Peroxisome proliferator-activated receptor Gamma Coactivator 1a, PGC-1 a; PhosphoInositide 3-Kinase, PI3K; PI3K Catalytic subunit type 3, PI3KC3; PhosphatidylInositol 4,5-bisPhosphate, PIP2; Peroxisome Proliferator-Activated Receptor, PPAR; Phosphatase and TENsin homolog, PTEN; PolyUnsaturated Fatty Acid, PUFA; protein REgulated in Development and DNA damage response 1, REDD1; Ras homolog enriched in brain, Rheb; Reactive Nitrogen Species, RNS; Reactive Oxygen Species, ROS; S6 Kinase, S6K; SKeletal Muscle, SKM; SoLute Carrier family 7 member 11, SLC7A11; Mothers Against Decapentaplegic, SMAD; spliced X-box Binding Protein 1, sXBP1; TriCarboxylic Acid, TCA; Transcription Factor A, Mitochondrial, TFAM; Transforming Growth Factor- b, TGF b; TGF b Receptor type-II, TGF b IIR; Toll-Like Receptor 4, TLR4; TNF Receptor, TNFR; Tumor Necrosis Factor-a, TNF- a; Tripartite motif containing 63, Trim63; Tuberous Sclerosis Complex, TSC; UnCoupling Protein, UCP; Unc-51 Like autophagy activating Kinase, ULK; Unfolded Protein Response, UPR; Ubiquitin-Proteasome System, UPS; b -HydroxyButyrate, b HB.

## CRediT authorship contribution statement

**Benjamin Hay:** Writing – original draft, Conceptualization. **Aurélien Brun:** Writing – original draft. **Anne Fougerat:** Writing – original draft, Conceptualization. **Vera Mazurak:** Writing – review & editing, Supervision, Project administration, Funding acquisition. **Olivier Le Bacquer:** Writing – review & editing, Supervision. **Jérémie Talvas:** Writing – review & editing, Supervision, Project administration, Conceptualization. **Frédéric Capel:** Writing – review & editing, Validation, Project administration, Funding acquisition, Conceptualization.

## Declaration of competing interest

The authors declare that they have no known competing financial interests or personal relationships that could have appeared to influence the work reported in this paper.
